# Evidence Supporting a Role of Alternative Splicing Participates in Melon (*Cucumis melo* L.) Fruit Ripening

**DOI:** 10.3390/ijms25115886

**Published:** 2024-05-28

**Authors:** Wenjiao Wang, Yuping Wei, Zhaoying Xu, Chengcheng Shen, Ang Li, Dailu Guan, Xuejun Zhang, Bin Liu

**Affiliations:** 1 College of Horticulture, Shanxi Agricultural University, Jinzhong 030801, China; weiyuping1227@163.com (Y.W.); shenchengcheng0608@163.com (C.S.); 2 Hami-Melon Research Center, Xinjiang Academy of Agricultural Sciences, Urumqi 830091, China; zhangxuejun@xaas.ac.cn; 3 College of Life Science and Technology, Xinjiang University, Urumqi 830046, China; liang013020@163.com; 4 Department of Animal Science, University of California Davis, Davis, CA 95616, USA; dguan@ucdavis.edu

**Keywords:** fruit ripening, alternative splicing, melon (*Cucumis melo* L.), serine/arginine-rich gene family

## Abstract

One key post-transcriptional modification mechanism that dynamically controls a number of physiological processes in plants is alternative splicing (AS). However, the functional impacts of AS on fruit ripening remain unclear. In this research, we used RNA-seq data from climacteric (VED, Harukei 3) and non-climacteric (PI, PS) melon cultivars to explore alternative splicing (AS) in immature and mature fruit. The results revealed dramatic changes in differential AS genes (DAG) between the young and mature fruit stages, particularly in genes involved in fruit development/ripening, carotenoid and capsaicinoid biosynthesis, and starch and sucrose metabolism. Serine/arginine-rich (SR) family proteins are known as important splicing factors in AS events. From the melon genome, a total of 17 SR members were discovered in this study. These genes could be classified into eight distinct subfamilies based on gene structure and conserved motifs. Promoter analysis detected various cis-acting regulatory elements involved in hormone pathways and fruit development. Interestingly, these *SR* genes exhibited specific expression patterns in reproductive organs such as flowers and ovaries. Additionally, concurrent with the increase in AS levels in ripening fruit, the transcripts of these *SR* genes were activated during fruit maturation in both climacteric and non-climacteric melon varieties. We also found that most *SR* genes were under selection during domestication. These results represent a novel finding of increased AS levels and *SR* gene expression during fruit ripening, indicating that alternative splicing may play a role in fruit maturation.

## 1. Introduction

Alternative splicing (AS) refers to the process whereby alternative splice sites are selected to create more than one transcript from heterogeneous nuclear RNA (hnRNA) transcripts. The spliceosome carries out this process, thereby increasing transcriptomic and proteomic diversity [1]. AS events are crucial for plant development and responses to the environment [2,3,4].

The spliceosome, a massive macromolecular complex made up of five small heterogeneous nuclear ribonucleoproteins (snRNPs) and several proteins, mediates the process [5]. Serine/arginine-rich (SR) proteins are essential splicing factors that bind to splicing enhancers on the pre-mRNA to control the selection of splicing sites [6]. Generally, SR proteins are considered activators, while heterogeneous nuclear ribonucleoproteins (hnRNPs) are regarded as repressors. In plants, SR proteins were initially identified in *Arabidopsis* [7], followed by their discovery in rice [8], maize [9], wheat [10], tomato [11], cassava [12], cotton [13], Brassica napus [14], and others [15]. One or more N-terminal RNA recognition motif (RRM) domains (PF00076) are found in typical SR proteins. These are followed by a downstream RS domain, which is composed of at least 50 amino acids and contains 40% RS and is distinguished by repeated RS or SR repetitions [16]. Typical SR proteins can be divided into six subfamilies, including SC, SCL, RS, SR, RSZ, and RS2Z, of which three subfamilies (SR, SCL, and RSZ) are plant-specific [17]. SC subfamily members have an RS domain after a single RRM domain, while members of the SCL (SC-like) subfamily have a short N-terminal extension with many RS and SR dipeptides. There are two RRM domains in the SR subfamily, and the second one has a conserved SWQDLKD motif. Members of the RSZ subfamily have one zinc knuckle (ZnK) domain and one RRM domain, while members of the RS2Z subfamily have one extra ZnK domain [17,18]. Additionally, two kinds of atypical SR proteins, known as SR45a and SR45, were discovered in earlier research. On either side of the RRM, SR45 includes two different RS domains, but SR45a encodes a protein that has both RS and RRM domains [7,19]. While the RS domain promotes protein-protein interactions, RNA-binding domains (RBDs) are responsible for recognizing and binding specific RNA sections. The molecular roles of SR proteins are intimately correlated with their subcellular location. They are said to be found in nuclear speckles, yet some of them can move back and forth between the cytoplasm and the nucleus [20].

Previous research has demonstrated that SR proteins modulate plant tolerance to abiotic stress. For example, plant calcium tolerance is decreased by *Atsr34B* by modulating IRT1 expression [21], while *AtRS40* and *AtRS41* play critical roles in salt stress modulation [22]. *GhSCL-8* positively regulates salt tolerance in cotton by responding to salt stress along with other *GhSR* genes [13]. Overexpression of *PtSCL30* in *Arabidopsis* reduces freezing and salt tolerance [23]. *SlSR1* and *SlSR3L*, two tomato *SR/CAMTA* transcription factors, adversely control the response to disease, but *SlSR1L* favorably regulates the ability to tolerate drought stress [24]. Under high temperatures in grapevines, *SR45*, *SR30*, and *SR34,* as well as the nucleus ribonucleic protein U1A, are likely to induce AS events [25]. The control of alternative splicing in response to light and temperature stress in vegetable crops, producing splicing variants with different functions with CRISPR/Cas9 technology, shows potential for improving vegetable crop performance in stressful conditions [26].

Conversely, SR proteins are crucial for the growth and development of plants. In *Arabidopsis*, proteins such as SC35 and SCL control silique phyllotaxy, root length, leaf development, and flowering time [27]. Fruit ripening in banana fruit is facilitated by the transcriptional repressive *MaMYB16L* (full-length isoform) being down-regulated and *MaMYB16S* (truncated isoform) being up-regulated [28]. The heat-resistant pakchoi cultivar, which is probably regulated by both transcriptional regulation and splicing factors, can reduce heat-induced leaf damage by inducing an unfolded protein response and preventing chloroplast growth and energy utilization [29]. The transcription level of numerous genes that are necessary for the synthesis of carotenoid in ripening tomato fruits is impacted by the deletion of RZ1AL since it has been demonstrated that some RZ members influence the splicing process of distinct genes [30]. Melon is a crucial economic and horticultural crop worldwide. Melon could be divided into two subgroups: *C. melo* subsp. Melo (hereafter referred to as Melo) and *C. melo* subsp. Agrestis (hereafter referred to as agrestis), is based on ovary pubescence. Knock-out of *CmNAC-NOR* affects melon climacteric fruit ripening [31]. Through DAP-seq analysis [32], we identified target genes of pre-mRNA processing splicing factors. Developmental mechanisms and the growing environment affect melon quality and production. While SR members’ functions in controlling many elements of development have been shown in numerous crops, nothing is known about how AS affects the ripening of melon fruit, and less is known about SR members in melon.

In the current research, we investigated alternative splicing (AS) throughout fruit development using RNA-seq data from immature and mature melon fruits. We conducted differential AS and expression analyses comparing young and mature fruits, and identified the melon *SR* gene family associated with AS regulation. Our findings revealed differential AS genes (DASGs) potentially influencing fruit quality in both climacteric and non-climacteric melon varieties. We identified 17 *CmSR* genes based on the melon genome using bioinformatics methods, analyzing their phylogeny and conserved domains. Despite prior attempts to integrate melon transcriptomes, a significant portion of melon RNA-Seq data remains unexplored. Here, we collected melon RNA-seq samples via the Illumina platform from the NCBI SRA database, allowing for a comprehensive global gene expression analysis across various samples, tissues, and cultivars. Utilizing the TPM matrix, we examined *CmSR* gene expression across different tissues and fruit developmental stages, providing novel perspectives on the functions, roles, and evolution history of the melon *CmSR* genes. The detailed AS analysis in melon not only enhances our understanding of climacteric and non-climacteric melon varieties but also provides valuable information for regulating fruit traits at the post-transcriptional level.

## 2. Results

### 2.1. Alternative Splicing Landscape Changes in Young and Mature Stages

**Figure 1:**
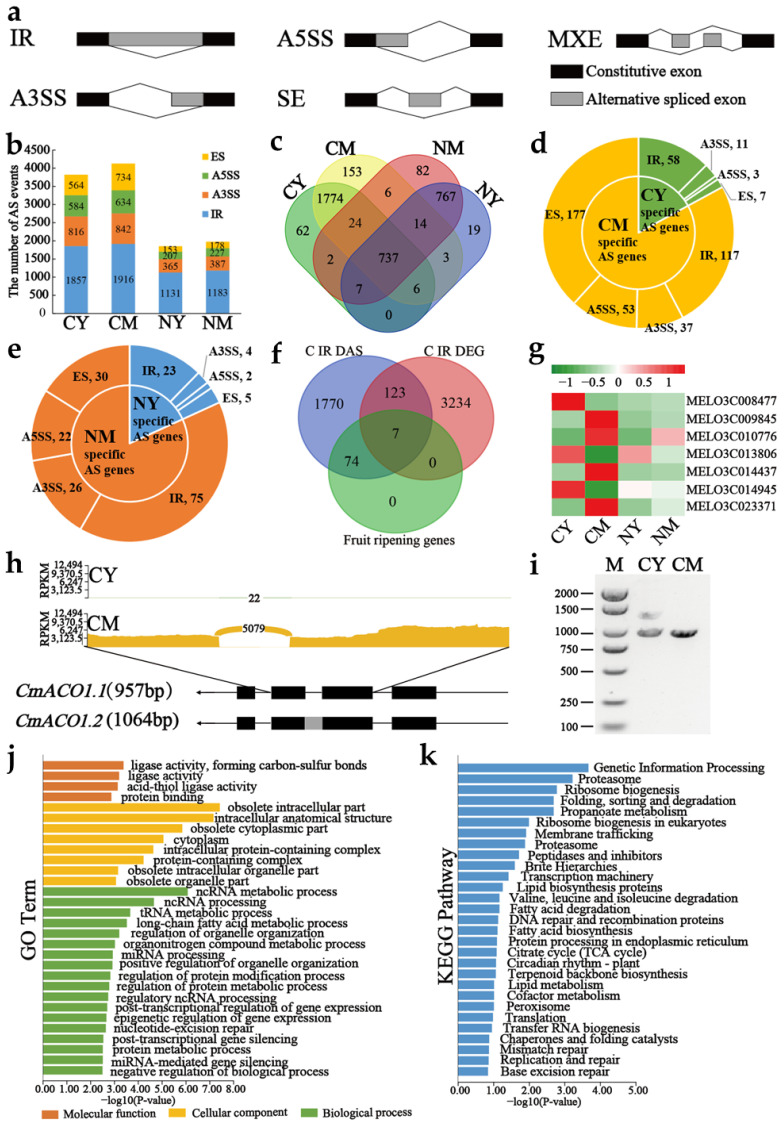
(**a**) Types of alternative splicing (AS). (**b**) The number of genes with AS at different stages. (**c**) Wenn diagram of genes splicing at different stages. (**d**) Distribution of stage-specific AS events in climacteric melon. (**e**) Distribution of stage-specific AS events in non-climacteric melon. (**f**) The Venn diagrams of DASGs that occur IR events, DEGs in CY and CM, and fruit-related genes that occur IR events. (**g**) The heatmap of seven fruit-related genes. (**h**) Sashimi plot showing the intron retention event of *CmACO1* (*MELO3C014437*). (**i**) Validation of the intron retention event in Figure 1h via reverse-transcription PCR (RT-PCR). M, Trans2K Plus DNA Marker. RT-PCR could amplify two bands: bright bands represent highly expressed transcripts, and dark bands represent low-expression transcripts. (**j**) Gene ontology (GO) of the common *AS* genes in climacteric melon and non-climacteric melon. (**k**) KEGG pathway of the common *AS* genes in climacteric melon and non-climacteric melon.

To identify alternative splicing (AS) events in melon, we analyzed a series of 18 melon fruit samples, comprising five biological replicates each of climacteric melon fruits at the young (CY) and mature stages (CM) and four biological replicates each of non-climacteric melon fruits at the young (NY) and mature stages (NM) (Table S1), to detect AS events. The phenotypic data for CY, CM, NY, and NM is shown in Figure S1. The melon reference genome ‘DHL92 v4’ comprises 28,299 genes, of which 73.80% (20,885) are multiexon genes. We explored five types of AS events, including IR, A5SS, A3SS, ES, and MXE (Figure 1a). According to our findings, intron retention (IR) was the most common type, accounting for 49% of events, followed by alternative 3′ splice site (A3SS) type (21%), alternative 5′ splice site (A5SS) type (15%), and exon skipping (ES) type (15%) AS events. Climacteric melon exhibited 3821 AS events in young fruit (CY) and 4126 AS events in mature fruit (CM), while non-climacteric melon showed 1856 AS events in young fruit (NY) and 1975 AS events in mature fruit (NM). Comparing climacteric and non-climacteric melon, the number of AS genes and events was highest in climacteric melon, indicating its greater protein diversity. In climacteric and non-climacteric melon, AS was dramatically more common in the mature period than in the young period (Figure 1b; Table S2).

The Venn diagram in Figure 1c illustrates the number of specific and common alternative splicing (AS) genes at the CY, CM, NY, and NM stages. Previous research has shown that in eukaryotes, stage-specific AS controls particular developmental processes [33]. Thus, we aimed to identify stage-specific *AS* genes during fruit development in both climacteric and non-climacteric melon. More stage-specific AS events were found in mature fruit compared to young fruit in climacteric melon (Figure 1d), consistent with previous research results [34]. A similar trend was observed in non-climacteric melon as well (Figure 1e), suggesting a specific pattern.

Additionally, we analyzed differentially expressed genes (DEGs) (Figure 1f). In the climacteric melon, 3364 DEGs and 1974 differentially alternative splicing (DAS) genes were identified. Among these, we selected 81 genes related to fleshy fruit ripening, including ethylene-responsive genes, abscisic acid-insensitive genes, genes involved in starch and sucrose metabolism, carotenoid and flavonoid biosynthesis, glycan biosynthesis and metabolism, and cysteine and methionine metabolism. The Venn diagram revealed minimal overlap (7 genes). Notably, *MELO3C014437* (*CmACO1*), *MELO3C009845* (*CmCGS*), *MELO3C010776* (*CmBCAT1*), and *MELO3C023371* (*CmGKC*) showed high expression levels in CM (Figure 1g). CmACO1, associated with ripening and ethylene biosynthesis [35,36,37], and *CmCGS*, a regulator of ethylene synthesis biochemical flux and SAM production [38], may contribute to reduced methylation in ACO1 antisense fruit. Additionally, ETH may control the synthesis of volatile aromatic and methyl-branched molecules through the relative expression of *CmBCAT1*, which catalyzes enhanced AT activity [39]. Furthermore, *CmGKC* is involved in plant photosynthetic carbon assimilation [40], suggesting a potential role in melon fruit ripening regulation.

We discovered a novel transcript for *CmACO1* that maintained an intron among the second and third exons among all intron retention (IR) events (Figure 1h). Additionally, *CmACO1* with an intron retained between the third and fourth exons was identified as having significant differential splicing. Similarly, differential splicing was observed for *CmACO1,* with an intron retained between the first and second exons. The isoform having intron retention is known as *CmACO1.2*, whereas the initial *CmACO1* transcript was identified as *CmACO1.1*. The coding parts of *CmACO1* from plants grown in CY and CM environments have been amplified and sequenced to confirm the existence of *CmACO1.2*. Upon RT-PCR amplification, two discrete bands were seen, signifying the preservation of every gene intron. Subsequent sequencing revealed amplicon lengths of 957 and 1292 bp, consistent with RNA-seq results, as depicted in Figure 1i. Additionally, confirmation of the new transcript was obtained by analyzing the bam file in the IGV tool. The validity of these AS events was further shown by the Sanger sequencing results of PCR products (Figure S2).

We performed GO and KEGG annotation analyses on 737 common alternative splicing (AS) genes throughout the CY, CM, NY, and NM phases to obtain a better understanding of the roles of these genes. The GO enrichment analysis revealed enrichment in the “protein modification process” and “mRNA processing” (Figure 1g). Furthermore, KEGG pathway enrichment analysis indicated involvement in pathways such as “Spliceosome”, “Glycan biosynthesis and metabolism”, “Fructose and mannose metabolism”, and “Pyrimidine metabolism” (Figure 1k). AS serves as necessary for regulating growth, development, flowering, stress responses, and other processes of biology in plants [41]. Therefore, it is not surprising that *AS* genes are linked to a variety of biological processes and engage in several pathways.

### 2.2. Identification of the CmSR Family

Plants have highly conserved serine/arginine-rich (SR) splicing factors, which regulate pre-mRNA splicing. *SR* genes were originally found in the melon database by using the Blastp program, based on *Arabidopsis* SR sequences, to identify all *SR* genes in the melon genome. Seventeen *CmSR* genes were identified in the melon genome. It was discovered that the predicted melon SR proteins ranged in length from 186 to 417 amino acids (AA), with a mean length of 272 aa. Their theoretical isoelectric points (pI) differed from 10.18 to 12.86 kDa, and their molecular weights (MW) were between 20.82 and 46.69 kDa (Table S3).

### 2.3. Phylogenetic Analysis, Syntenic Analysis, Gene Structure and Conserved Motifs of the CmSR Family

**Figure 2:**
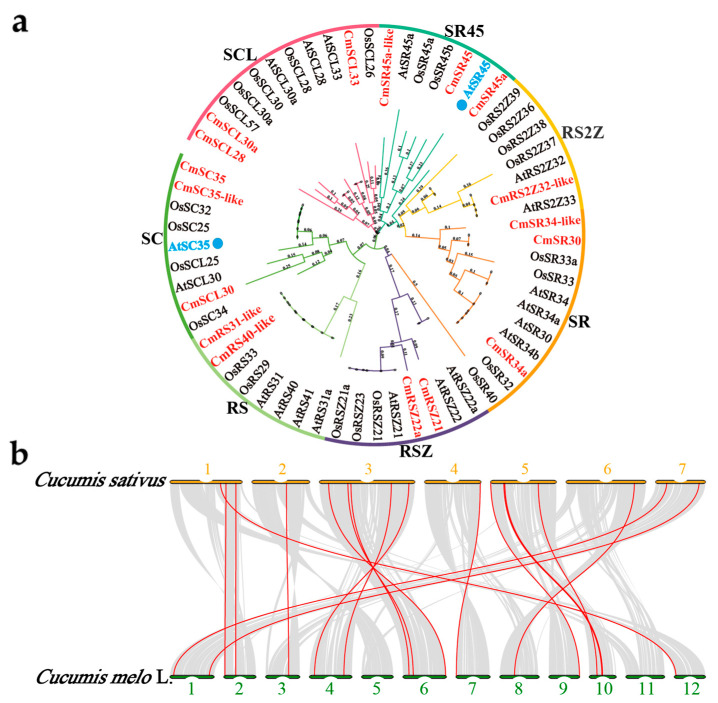
(**a**) Phylogenetic analysis of SR proteins in *Arabidopsis thaliana*, *Oryza sativa*, and *Cucumis melo* L. All SR proteins were grouped into seven subfamilies, with a distinct color assigned to each subfamily. (**b**) Synteny between the genomes of *Cucumis sativus* and *Cucumis melo* L. Grey lines represent syntenic blocks between genomes, and red lines indicate syntenic *SR* gene pairs.

We performed phylogenetic analysis on 17 *CmSR* genes to understand their evolutionary relationships among *Arabidopsis thaliana*, *Oryza sativa*, and *Cucumis melo* L. based on their protein sequences. Seventeen *CmSR* genes were finally clustered into eight subfamilies (Figure 2a). The earlier identifying system, which included the subfamilies SR, SCL, RS2Z, RSZ, RS, SR45, SR45a, and SC, was also utilized in this work. The biggest subfamily was SCL, which had four *SR* genes. The smallest subfamily was SR45, which had one *SR* gene. The remaining subfamilies were SR, SC, RS, RSZ, SR45, and RS2Z, which had three, two, two, one, and one *SR* gene each. Despite belonging to a family, *CmSR* genes exhibit considerable variation in basic information. Phylogenetic analysis revealed a consistent relationship between classification and evolution.

The chromosomal locations of *CmSR* genes were analyzed using the MG2C online website [42], revealing their irregular distribution across ten chromosomes (Table S3). The distribution of *CmSR* genes on melon chromosomes is as follows: one gene each on chromosomes 3 (*CmSC35-like*), 7 (*CmSR45*), 8 (*CmSR45a*), 9 (*CmSR34a*), and 12 (*CmSR45a-like*); two genes each on chromosomes 1 (*CmSC35* and *CmSR34-like*), 2 (*CmSR30*, *CmSCL30a*), and 4 (*CmRSZ22a*, *CmSCL33*); three genes on chromosomes 6 (*CmRS31-like*, *CmSCL28*, and *CmRS40-like*) and 10 (*CmRSZ21*, *CmRS2Z32-like*, and *CmSCL30*); and no genes were found on the other two chromosomes.

The genus *Cucumis* (family Cucurbitaceae) comprises two globally cultivated economically important vegetable crop species: cucumber (*Cucumis sativus* L., 2*n* = 14) and melon (*Cucumis melo* L., 2*n* = 24). Additionally, both melon and cucumber are believed to have originated in Asia, deriving from a common ancestor approximately nine million years in history [43]. To learn more about the melon SR family’s phylogenetic mechanisms, we constructed two comparative syntenic maps of *Cucumis melo* with representative species of the genus *Cucumis* (Figure 2b). A total of 17 *SR* genes exhibited a syntenic relationship with those in *Cucumis*. Figure 2b showed that melon Chromosome I was syntenic to cucumber Chromosome 7. Moreover, Chromosomes II were syntenic with cucumber Chromosome 1; Chromosomes III were syntenic with cucumber Chromosome 2; Chromosomes IV and VI were syntenic with cucumber Chromosome 3; Chromosomes VII were syntenic with cucumber Chromosome 4; Chromosomes VIII were syntenic with cucumber Chromosome 6; and Chromosomes IX and X were syntenic with cucumber Chromosome 12, comparable to studies in cucumbers [44]. Collectively, those syntenic patterns suggest a complicated history with chromosomal structural alterations, including rearrangement, loss, and fusion, during melon evolution.

**Figure 3:**
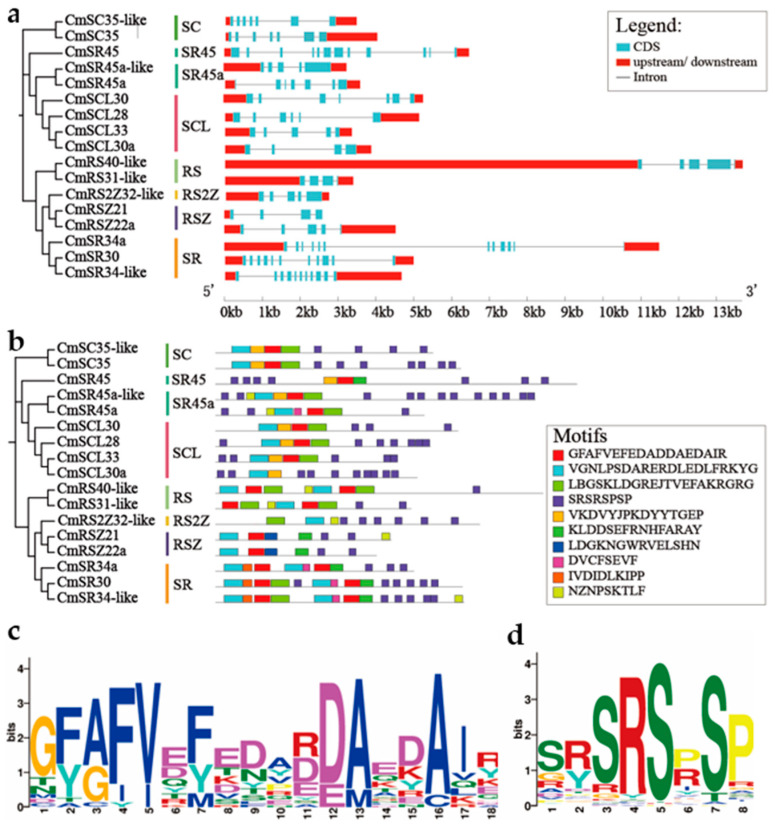
(**a**) *SR* gene exon/intron structure. Exons are shown by blue boxes, introns by black lines, and UTRs by red boxes. (**b**) The distribution of SR protein conserved motifs. Ten motifs are indicated by colored boxes. (**c**) TBtools’ software (v2.069) was used to investigate the RRM conserved domains’ seq-logo. Highly conserved residues were represented by different colors. These residues are G, F, V, F, V, F, D, A. (**d**) The TBtools software (v2.069) was used to assess the seq-logo of the RS or SR repeat domains. Highly conserved residues were represented by different colors, among them, S, R, S, R, S, and P.

Gene structure is essential to comprehending the relationship between genomic evolution and functional differentiation in members of multigenic families. To explore the structural diversity of *CmSR* genes, we constructed an exon-intron map based on their genomic and coding sequences (Figure 3a). While the number of exons varied substantially across members of the SCL subfamily (4~7 exons), the gene structure (exon and intron) of *SR* genes was largely similar throughout the RSZ (4–5 exons), RS2Z (5 exons), SR45 (12 exons), SR45a (5~7 exons), SR (11 exons), and SC (7 exons) subfamilies. *CmSR* genes exhibit significant variation in the number and length of exons and introns, suggesting that the CmSR family may play specific or redundant roles in evolution.

Ten motifs were identified in CmSR proteins, with each subfamily exhibiting a conserved motif pattern (Figure 3b). Motif 1 (Figure 3c) and motif 2 are recognized by the RRM_1 domain, and motif 4 is rich in arginine and serine dipeptides (Figure 3d). Motif 1 and motif 2, along with motif 4, were present in every SR protein. Motif 6 was found exclusively in SR and RSZ, motif 7 exclusively in RSZ, motif 8 exclusively in SR and SR45a, and motif 9 exclusively in SR. The structure of changes among various groups, along with the conserved motifs across groups, suggests diverse functions within the *CmSRs*.

### 2.4. Cis-Elements in Promoter Regions and Gene Expression Analysis of SR Proteins

**Figure 4:**
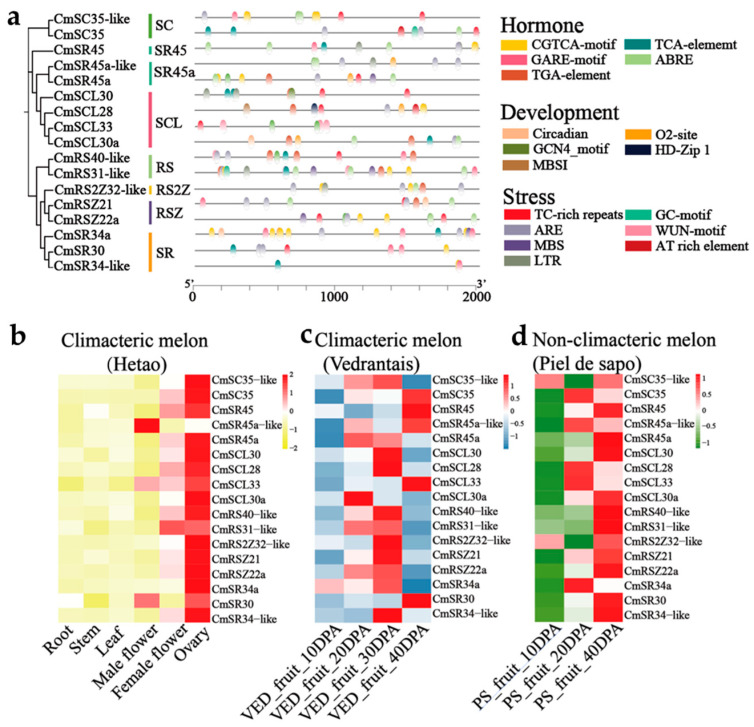
(**a**) Predicted cis-elements in the *CmSR* gene promoter regions. Various colors are used to indicate distinct cis-elements. (**b**) Expression heatmap of *CmSR* genes in different tissues using TPM. (**c**) Expression heatmap of *CmSR* genes in different fruit development stages of climacteric melon VED using TPM. (**d**) Expression heatmap of *CmSR* genes in different fruit development stages of non-climacteric melon PS using TPM. The colors, which vary from blue to red and from yellow to red, represent the scales of the relative expression levels.

Cis-elements in the promoter areas of *CmSR* genes, which regulate how plants develop and grow, influence the expression of target genes. It is essential for studying the cis-acting elements in the CmSR family’s promoter regions. The findings of the analysis indicated that the promoters of *SR* genes contained cis-acting regulatory elements associated with development, stress, and hormones (Figure 4a; Table S4). ARE elements, which are necessary for anaerobic induction, were present in the majority of *SR* genes (14/17, 94.92%). Hormone-responsive and stress-related cis-elements were detected, including ABA-responsive (ABRE, 13/17, 76.47%), MeJA-responsive (CGTCA-motif, 10/17, 58.82%), salicylic acid-responsive (TCA, 9/17, 52.94%), gibberellin-responsive (GARE, 8/17, 47.06%), and auxin-responsive (TGA, 8/17, 47.06%) elements. Additionally, developmental elements were detected, including those involved in zein metabolism regulation (O2-site, 5/17, 29.41%), circadian control (circadian, 3/17, 17.65%), endosperm expression (GCN4_motif, 1/17, 5.88%), differentiation of palisade mesophyll cells (HD-Zip1, 1/17, 5.88%), and regulation of flavonoid biosynthetic genes (MBSI, 1/17, 5.88%). Stress-responsive elements, including defense and stress responsiveness (TC-rich repeats, 10/17, 58.82%), low-temperature responsiveness (LTR, 5/17, 29.41%), and drought-inducibility (MBS, 4/17, 23.53%), were also prevalent in *SR* gene promoters. The autocatalytic synthesis of abscisic acid and its cooperative connection with auxin regulate fruit ripening [45]. The findings suggest that a large number of *SR* genes in melon are connected to the development, growth, and stress response of plants.

We used RNA-Seq data from six tissues of the climacteric melon “Hetao” to investigate the expression patterns of *CmSR* genes in order to anticipate their possible roles (Figure 4b). The findings showed that there was a considerable variation in the *CmSR* gene expression levels between different tissues. *CmSR45a-like* and *CmSR30* exhibited high expression levels in male flowers but low levels in female flowers. Apart from *CmSR45a-like* and *CmSR30*, the *CmSR* genes were found to be strongly expressed in the ovary and to be lowly expressed in the root, indicating that they may be involved in controlling fruit development. We investigated the expression of *CmSR* genes at different stages of fruit development (Figure 4c,d). The results indicated that *CmSC35*, *CmSR45a-like*, *CmSCL28*, *CmSCL33*, and *CmSR34a* were highly expressed during the mature period in VED, whereas the opposite trend was observed in PS. *CmSCL30a* was highly expressed during the young period in VED, whereas the opposite trend was observed in PS. *CmRS31-like* and *CmRSZ22a* were highly expressed during the mature period in PS and exhibited high expression levels in both young and mature stages. *CmSR45*, *CmSCL30*, *CmRS40-like*, *CmRS2Z32-like*, *CmRSZ21*, *CmSR30*, and *CmSR34-like* were highly expressed during the mature period in both climacteric melon (VED) and non-climacteric melon (PS), indicating that these *SR* genes may be important for the growth and development of fruit as well as for its early development.

**Figure 5:**
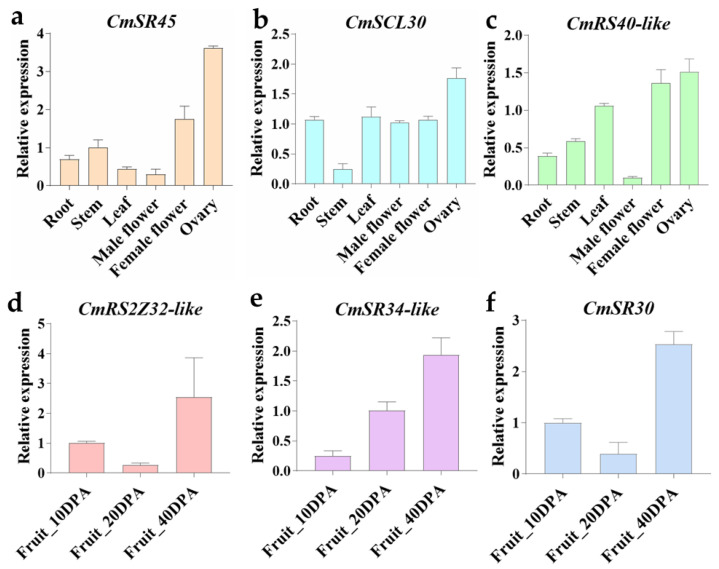
The expression level of *CmSR* genes under different tissue and different fruit development stages. qRT-PCR verified the expression of (**a**) *CmSR45*, (**b**) *CmSCL30*, and (**c**) *CmRS40-like* in different tissues of climacteric melon Hetao. QRT-PCR verified the expression of (**d**) *CmRS2Z32-like*, (**e**) *CmSR34-like*, and (**f**) *CmSR30* in different fruit development stages of climacteric melon VED.

To validate the expression level of *CmSR* genes obtained from RNA-seq, a qRT-PCR assay was performed. As shown in Figure 5, we studied the expression of three genes (*CmSR45*, *CmSCL30,* and *CmRS40-like*) in different tissues of climacteric melon Hetao. The results showed a similar tendency with the RNA-seq data; it was discovered that *CmSR45*, *CmSCL30,* and *CmRS40-like* were lowly expressed in the root and highly expressed in the ovary, suggesting that they may be involved in regulating fruit development. *CmRSZ32-like*, *CmSR34-like,* and *CmSR30* were highly expressed in climacteric melon VED mature fruit stages, suggesting these which might be the key players and potential candidate genes in the development of melon fruit.

**Figure 6:**
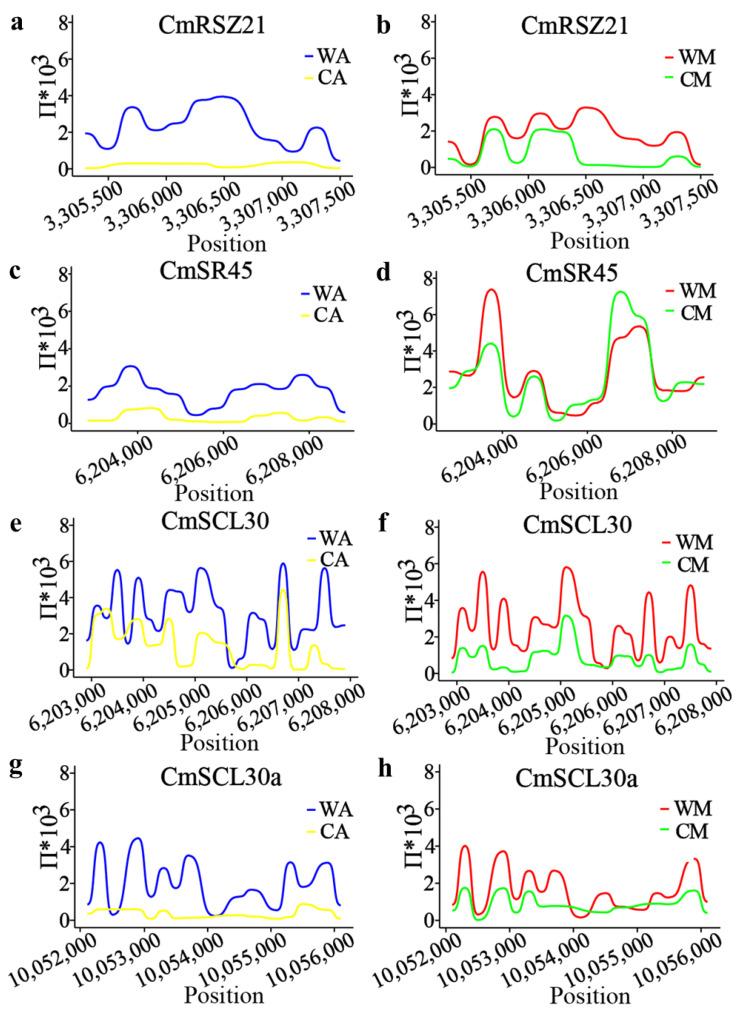
Nucleotide diversity of two melon populations (WA, CA) at individual nucleotide sites in (**a**) *CmRSZ21*, (**c**) *CmSR45*, (**e**) *CmSCL30,* and (**g**) *CmSCL30a*. Nucleotide diversity of two melon populations (WM, CM) at individual nucleotide sites in (**b**) *CmRSZ21*, (**d**) *CmSR45*, (**f**) *CmSCL30,* and (**h**) *CmSCL30a*. The *x*-axis indicates the genome position in the melon (DHL92) v4 genome, while the *y*-axis shows each melon group’s π value.

Due to the extensive genomic and functional diversity of *CmSR* genes, we investigated whether they underwent selection. To address this question, we scanned published genomic sequences from 1165 melon accessions for evidence of selection [46]. Specifically, we observed selection in the region surrounding *CmRSZ21* during melon domestication in both *C. melo* ssp. agrestis and *C. melo* ssp. melo (Figure 6a,b). *CmSR45*, *CmSR34-like*, *CmSC35-like*, *CmSCL33*, *CmSCL28*, *CmRS40-like*, *CmSR45a*, *CmSR34a*, *CmSR30*, and *CmSC35* were selected in *C. melo* ssp. agrestis during melon domestication, whereas the opposite trend was observed in *C. melo* ssp. melo (Figure 6c,d). *CmSCL30* was selected in *C. melo* ssp. melo during melon domestication, while the opposite trend was shown in *C. melo* ssp. agrestis (Figure 6e,f). *CmSCL30a*, *CmRSZ22a*, *CmRS31-like*, *CmRS2Z32-like*, and *CmSR45a-like* were not selected during melon domestication in either *C. melo* ssp. agrestis or *C. melo* ssp. melo (Figure 6g,h).

## 3. Discussion

In line with prior research, we observed a significant increase in transcriptome diversity due to alternative splicing (AS) in plants with fruits, with approximately 20% of genes undergoing alternative splicing. Since alternative splicing (AS) exhibits species and stage specificity [34], we compared AS events between young and mature stages in both climacteric and non-climacteric melon fruits. The primary event was consistently intron retention (IR), and there was a significant variation in the number of AS events among young and mature periods. Alternative splicing at different stages was seen in certain genes related to fruit ripening. For instance, all introns of *CmACO1* are retained during the young fruit stage. Alternative splicing generates two isoforms of *CmACO1*: the highly expressed *CmACO1.1* (957 bp) and the low-expressed *CmACO1.2* (1292 bp). We hypothesize that intron retention (IR) occurs in *CmACO1* during the young fruit stage, resulting in the formation of *CmACO1.2*, which suppresses the expression of the *CmACO1.1* transcript. In the mature fruit stage, alternative splicing is absent, leading to a high expression of *CmACO1.1*, which promotes fruit ripening. This observation reveals the regulatory role of alternative splicing in fruit ripening. Therefore, further investigation with additional developmental stages and species is warranted to confirm whether this pattern is generalizable or species-specific. A crucial part of plant development and growth is played by alternative splicing [47]. Through alternative splicing, plants protect against the accumulation of *CsGA2ox8* transcripts caused by intense light stress. They do this by precisely regulating gibberellin levels to preserve hypocotyl elongation [48]. Splicing factors are necessary for splicing to be executed and regulated. Among these factors, serine/arginine-rich (SR) proteins serve as splicing enhancers, promoting the utilization of suboptimal splice sites [6]. 

Recently, genome-wide studies have investigated SR proteins in many plants to elucidate their evolution and function. Initially identified in *Arabidopsis* [17], SR proteins have since been discovered in various plants, including rice [8], maize [9], wheat [10], tomato [11], cassava [12], cotton [13], Brassica napus [14], and others [15]. Based on entire sequences or domain sequences, five to seven subfamilies of *SR* genes were identified. This work discovered and described 17 melon *SR* genes. A comprehensive examination of *SR* genes was carried out, encompassing the relationships of evolution among *Arabidopsis thaliana*, *Oryza sativa*, and *Cucumis melo* L. Eight subfamilies were also identified based on the 17 *SR* genes found in melon. Melon (6/17, 35.30%) has a similar percentage of plant-specific subfamily members to other plants [10]. Some biological activities have been shown to include the *Arabidopsis* splicing factor serine/arginine-rich 45 (SR45). The sr45-1, through a loss-of-function mutant has abnormal metallic allocation, shorter siliques with fewer seed sets, slower growth of the roots, late blooming, and an odd number of floral organs [49]. Mutations in SC35 and SC35-like (SCL) proteins can cause pleiotropic changes in lant shape and development. These modifications consist of shorter roots, delayed blossoming, serrated leaves, and abnormal silique phyllotaxy [27], so we predicted that the *CmSR45* subfamily and *CmSC* subfamily genes may participate in the melon fruit development process. Genes within the same subfamily typically share the same gene structures and conserved motifs. However, there are significant variances in cis-acting regulatory elements in promoters, which could influence expression patterns [50,51]. Given that the GCN4_motif and MBSI motif are associated with fruit development, we predict that *CmSCL28* and *CmSCL30* may participate in the melon fruit development process.

We exclusively analyzed RNA-seq data originated by the Illumina platform in the SRA database. 943 RNA-seq samples in total were obtained from the NCBI database. After applying various filtering criteria, we obtained a high-quality TPM matrix comprising 864 samples. In RNA-seq analyses of various tissues, *SR* genes were significantly more highly expressed in female flowers and ovaries compared to roots, stems, and leaves, likely reflecting their crucial role in fruit development. In mature stages, we found that several *SR* genes expressed at reduced levels, suggesting that pre-mRNA processing regulators are more important during cell growth in early green fruits [11]. In both climacteric melon VED and non-climacteric melon PS, *CmSR45*, *CmSCL30*, *CmRS40-like*, *CmRS2Z32-like*, *CmRSZ21*, *CmSR30*, and *CmSR34-like* were highly expressed at 40 DPA, suggesting their potential involvement in fruit ripening regulation. We observed reduced nucleotide diversity (π) in the genomic regions surrounding *CmSR30* (chr02: 620437-625412(−)) and *CmSR45* (chr07: 6202616-6209077(+)) in domesticated melon groups (*C. melo* L. ssp. agrestis (CA) compared with *C. melo* L. var. agrestis (WA), indicating the potential selection of *CmSR30* during melon domestication. However, further investigation into the biological function of *CmSR* genes is necessary in the future.

## 4. Materials and Methods

### 4.1. Plant Materials and Data Source

Projects involving transcriptome sequencing were found by searching the NCBI SRA database (https://www.ncbi.nlm.nih.gov/sra), and the Run Selector tool was utilized to extract information. Technically problematic samples were removed, including (i) empty FASTQ files, (ii) paired-end samples with single-end reads, and (iii) paired-end reads with uneven lengths. In total, we downloaded 943 raw RNA sequencing data SRA files (accessed on 1 October 2022) using Kingfisher (v0.1.2) and decompressed them using the SRA-TOOLKIT (v.2.5.7) [52]. 

### 4.2. Identification of AS Genes and Events

Climacteric melon at 10–20 days post anthesis (DPA) was described as a young stage, and at 40–50 DPA, it was described as a mature stage. Non-climacteric melon at 20 DPA was described as young, and at 40 DPA it was described as mature. AS events were detected using rMATS (v.4.2.0) [53] and included the following splicing events: alternative 3′ splice junction, alternative 5′ splice junction, exon skipping, intron retention, and mutually exclusive exons. The alignment was performed using the STAR aligner (v2.7.8a) with local mapping and in double-pass mode [54]. All alternative splicing events were normalized to the total transcript count in order to correct for total transcript levels. The following options were used: -t paired --readLength 150 --variable-read-length --allow-clipping --novelSS --mil 20 --mel 10321. The –novels option was utilized due to the absence of splicing variants reported in the DHL92 v4 annotation. A false discovery rate (FDR) of ≤0.05 was employed to identify significant differences in splicing events. GO and KEGG annotation data for every melon gene were obtained using eggNOG-mapper (http://eggnog-mapper.embl.de/ (accessed on 18 September 2023)), GO and KEGG enrichment analysis and visualization were performed using TBtools [55].

### 4.3. Validation of AS Events

PrimeScriptTM RT Master Mix (Takara, RR036A, Beijing, China) was used to generate cDNA after total RNA was extracted using a TIANGEN RNAprep Pure Plant Kit (Beijing, China) in accordance with the instructions. Using an ultra-trace UV-visible spectrophotometer (NanoVueTM Plus, Wilmington, DE, USA), the cDNA was examined for quality and concentration and maintained in reserve.

For RT-PCR, a 20 µL reaction volume was used, and each gene’s primer pair was designed to amplify both of its two splice variants (isoforms 1 and 2) in a single reaction. Table S5 lists the primers used in the RT-PCR study.

### 4.4. Identification of SR Genes in Cucumis melo L.

Melon genome sequence data *(Cucumis melo* L., cv. DHL92 v4 Genome) released at the Cucurbit Genomics Data website were used for the discovery and study of the melon *SR* gene [56]. From earlier research, typical and atypical SR proteins from *Oryza sativa* and *Arabidopsis thaliana* were obtained [57,58]. To find SR proteins in melon, a BLASTp search (e-value = 1 × 10^−10^) was performed using these SR proteins from model plants as queries [59]. Prior research served as the foundation for the definition of SR proteins. Specifically, sequences were submitted to the PfamScan (https://www.ebi.ac.uk/Tools/pfa/pfamscan/) website (accessed on 10 August 2023) to verify the presence of the RRM domain (PF00076) in the N-terminal, and the region downstream of the RRM domain contained an RS domain with a minimum length of 50 amino acids and at least 20% RS or SR dipeptides [60]. The protein properties of SR proteins were predicted by the ProtParam module in Biopython.

### 4.5. Phylogenetic Analysis of SR Family Members, Gene Duplication Analysis

To elucidate the evolutionary relationships between SR families, multiple alignments of SR amino acid sequences from *Arabidopsis thaliana*, *Oryza sativa*, and *Cucumis melo* L. were performed using ClustalW [61]. The neighbor-joining (NJ) approach with pairwise deletion, Poisson correction, and 1000 bootstrap analyses for CmSR proteins was used to build the phylogenetic tree. Evolutionary analyses were conducted using MEGA7 (version 7.0) and visualized on the iTOL website [62]. The locations of *SR* genes were retrieved from the annotation of the melon genome. Using BLASTP to align sequences at an e-value of 1e^−10^, gene duplication events were found. MCScanX was then used to discover duplication patterns, including segmental and tandem duplications. The visualization of duplication events was performed using the Circos software (v0.52) [63].

### 4.6. Analysis of Gene Structure, Conserved Motifs, and cis-Acting Regulatory Elements

Melon exon/intron position data was taken from the GFF/GTF files. To find motifs, whole protein sequences were uploaded to the MEME website [64], and their functions were verified using PFAM and SMART [65,66]. To predict cis-acting elements, cis-elements from the genomic DNA sequences 2000 bp upstream were submitted to the PlantCARE website and viewed with TBtools [55,67].

### 4.7. Preprocessing, Quality Control, and RNA-seq Data Analysis

Fastp was used to locate and eliminate adapters and poor-quality bases (v0.23.2). Too short, or too many N, quality lower than 20, adapters FASTQ files were considered to have insufficient quality and were removed. STAR (v2.7.8a) [54] was selected to perform alignment following quality control. Based on alignments from STAR, StringTie (v2.2.1) [68] was used for analyzing the levels of gene and transcript expression. 

### 4.8. QRT-PCR Analysis and Statistical Analysis

For qRT-PCR, the *CmACTIN* gene (GenBank: AY859055.1) was used as an internal control. Using the LightCycler480 real-time PCR detection system (Roche Diagnostics, Indianapolis, IN, USA), QRT-PCR detections for the target genes were carried out using the SYBR Green FAST Mixture qPCR kit (GenStar, Shenzhen, China). This was followed by a PCR program of 95 °C for 30 s, 40 cycles of 95 °C for 15 s and 60 °C for 10 s, and finally 72 °C for 30 s. Using the 2^−∆∆CT^ approach, the expression levels of each candidate gene were ascertained, and the relative transcript levels were computed and normalized in accordance with the previously mentioned procedures [69]. Table S6 has a list of all the primers that were utilized in this study. For every experiment, at least three biological replicates were used. Results are presented using GraphPad Prism 8 as means ± SD [70].

### 4.9. Analysis of Nucleotide Diversity

Resequencing data from 1165 published melon accessions (377 CA, 661 CM, 93 WA, and 34 WM) were used for the analysis [46]. Nucleotide diversity was estimated as π values for each population using VCFtools with the parameter –window-pi 500, comparing between the two groups (CA, WA, CM, WM) [71].

## 5. Conclusions

Transcriptome datasets from Illumina platforms revealed significantly more AS events in mature fruit compared to young fruit, indicating the potential participation of alternative splicing in regulating melon fruit ripening. The *SR* genes in melon were thoroughly identified and characterized across the whole genome. Eight subfamilies were identified from the identification of 17 *SR* genes in total. Genes that belong to the same subfamily have equivalent motifs and gene structures. Numerous cis-elements in the promoter region of *CmSRs* respond to fruit development. Expression patterns in various tissues and different fruit development stages revealed the important roles of *SR* genes in fruit development. These findings suggest that AS could be an important regulatory mechanism for melon fruit ripening. To sum up, our results give detailed information about *SR* genes in melon, providing a foundation for more functional research and offering candidate genes for improving melon fruit development.

## Data Availability

Data is contained within the article and Supplementary Materials and further inquiries can be directed to the corresponding author.

## Supplementary Materials

The following supporting information can be downloaded at: https://www.mdpi.com/article/10.3390/ijms25115886/s1.
