# Supplementary material for: Evidence Supporting a Role of Alternative Splicing Participates in Melon (Cucumis melo L.) Fruit Ripening
Source: Int J Mol Sci. 2024 May 28;25(11):5886. doi: 10.3390/ijms25115886 (PMC11172951; doi:10.3390/ijms25115886)
Supplement: Supplementary file 1 [file ijms-25-05886-s001.zip › ijms-2999705-SI/Supplemental Figure S1-S2.pdf]

Supplemental figure:

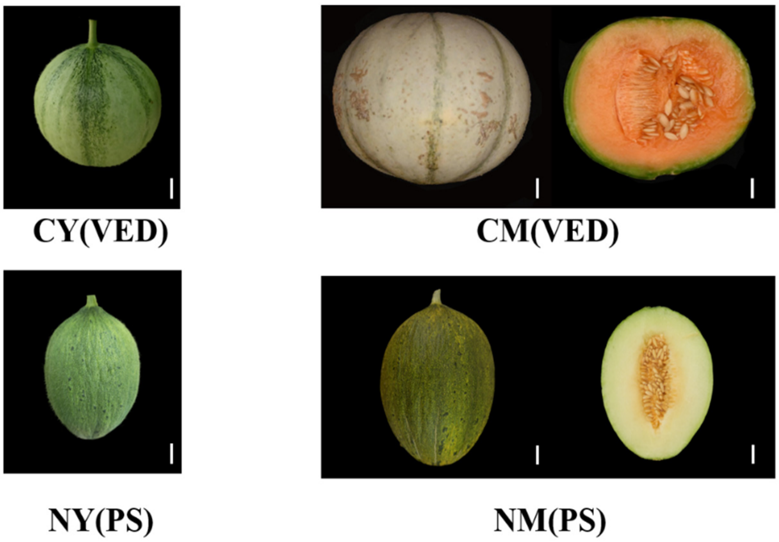

Figure S1. CY: climacteric melon young fruits; CM: climacteric melon mature fruits; NY: non-climacteric melon young fruits; NM: non-climacteric melon young fruits. Representative fruits of the CY, CM, NY, NM are shown. Scale bars, 1.0 cm.

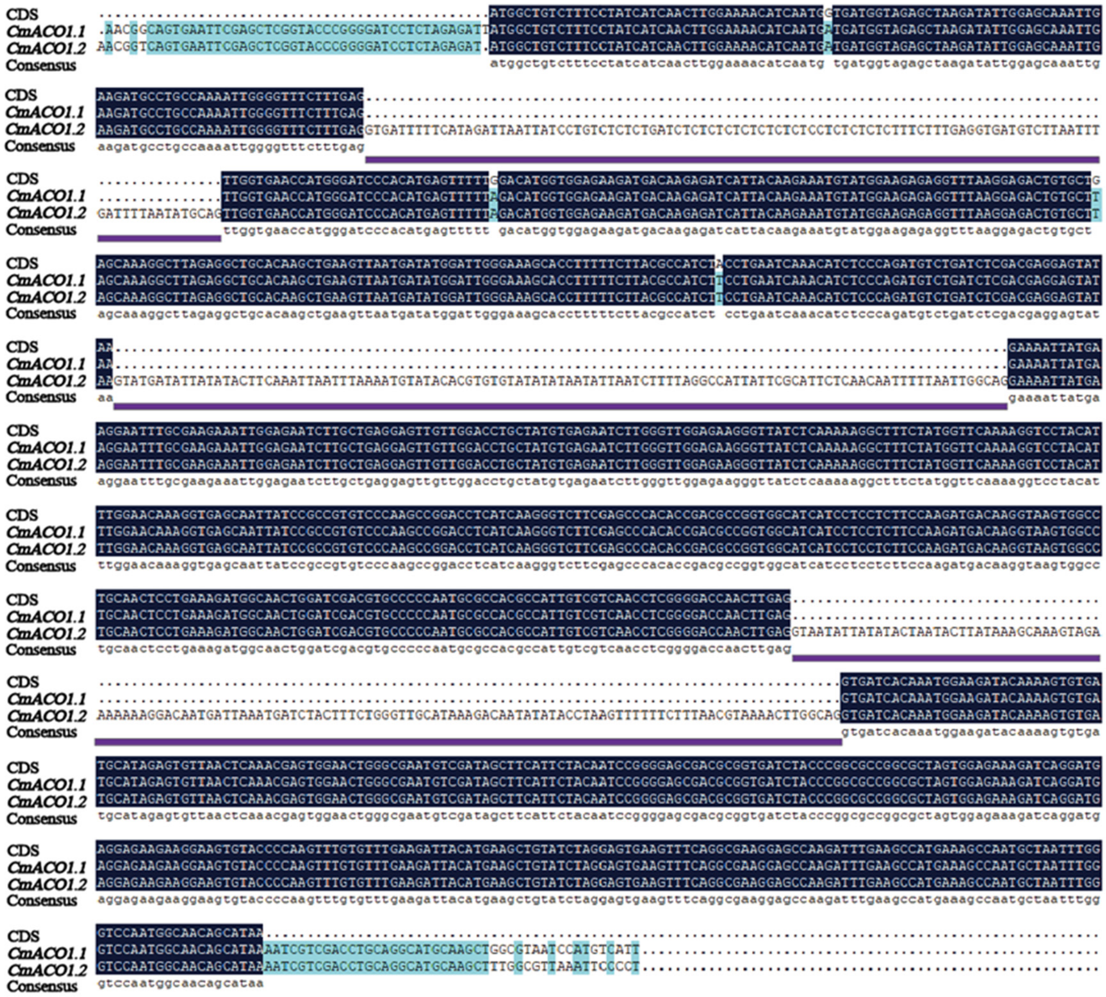

Figure S2. The alignment of the AS gene *CmACO1* Sanger sequencing results. The two transcripts' different splicing is shown by the purple line.
